# Supplementary material for: In Vitro Skeletal Muscle Model of PGM1 Deficiency Reveals Altered Energy Homeostasis
Source: Int J Mol Sci. 2023 May 4;24(9):8247. doi: 10.3390/ijms24098247 (PMC10179458; doi:10.3390/ijms24098247)

# In Vitro Skeletal Muscle Model of PGM1 Deficiency Reveals Altered Energy Homeostasis

## SUPPLEMENTARY MATERIAL

Supplementary Table S1 .................................................................................. pag. 2

Supplementary Table S2 .................................................................................. pag. 2

Supplementary Table S3 .................................................................................. pag. 2

Supplementary Table S4 .................................................................................. pag. 3

Supplementary Table S5 .................................................................................. pag. 3

Supplementary Table S6 .................................................................................. pag. 3

Supplementary Table S7 .................................................................................. pag. 5

Supplementary Table S8 .................................................................................. pag. 6

Supplementary Figure S1 .................................................................................. pag. 7

Supplementary Figure S2 .................................................................................. pag. 8

Supplementary Figure S3 .................................................................................. pag. 9

Supplementary Figure S4 .................................................................................. pag. 12

Supplementary Figure S5 .................................................................................. pag. 13

Supplementary Figure S6 .................................................................................. pag. 14

**Supplementary Table S1.** List of sgRNAs tested to generate *Pgm1*-KO C2C12 myoblasts.

| Gene | Ensembl Gene ID | Transcript | Exon | sgRNA no. | Primer sequences | Clone selected?* |
|---|---|---|---|---|---|---|
| *Pgm1* | ENSMUSG00000025791 | NC_000070.6 | Exon 2 | 1 | CACCGcctatcctagattggtcgcc | Yes Clone 1 |
| | | | | | AAACggcgaccaatctaggataggC | |
| | | | Exon 2 | 2 | CACCGaataaccaggcgaccaatct | Yes Clone 2 |
| | | | | | AAACagattggtcgcctggttattC | |
| | | | Exon 4 | 3 | CACCGcatgcacggaggtacgcgct | No |
| | | | | | AAACagcgcgtacctccgtgcatgC | |

* *Clone selection based on Pgm enzyme activity assay. Only KO clones with Pgm activity reduced to less than 5% of the activity in detected in the wild-type line were selected, as comparable with the levels detected in patients.*

**Supplementary Table S2.** List of primers using for Sanger sequencing of *Pgm1*-KO clones.

| Gene | Ensembl Gene ID | Exon | Primer sequences |
|---|---|---|---|
| *Pgm1* | ENSMUSG00000025791 | Exon 2 | [Fw] tgtaaaacgacggccagtACATGCACTGTGTTCTTGCC |
| | | | [Rv] caggaaacagctatgaccAGGGAAGCAAAACCCCTTCT |
| | | Exon 4 | [Fw] tgtaaaacgacggccagtACAGCTGTCACAGTAGCCCT |
| | | | [Rv] caggaaacagctatgaccGAATATGGGTGACCCGCTGA |

**Supplementary Table S3.** List of off-targets identified by online tools CHOPCHOP (https://chopchop.cbu.uib.no/) and CRISPOR (http://crispor.tefor.net/) for sgRNA1 and sgRNA2.

| **sgRNA 1** | | | | | |
|---|---|---|---|---|---|
| *19 predicted off-targets, of which 2 present 3 or less mismatches:* | | | | | |
| Mismatches | Location / Gene | Chromosome range | Sequence* | Presence of PAM seq (NGG) | Conclusion** |
| **0** | **Exon 2 / *Pgm1*** | **chr4: 99818643-99818662** | **CCTATCCTAGATTGGTCGCC** | **Yes (TGG)** | **On-target, gene of interest** |
| 3 | Intron 3 / *Abcc8* | chr7:45824707-45824729 | CCTAGCCTGGATTGGTCGCC | Yes (TGG) | Considered and assessed |

| **sgRNA 2** | | | | | |
|---|---|---|---|---|---|
| *53 predicted off-targets, of which 6 present 3 or less mismatches:* | | | | | |
| Mismatches | Location / Gene | Chromosome range | Sequence* | Presence of PAM seq (NGG) | Conclusion** |
| **0** | **Exon 2 / *Pgm1*** | **chr4: 99818651-99818670** | **AATAACCAGGCGACCAATCT** | **Yes (AGG)** | **On-target, gene of interest** |
| 2 | Intron 2 / *Cdh11* | chr8:103425462-103425484 | AATAACCAGGGGCCCAATCT | No (TAG) | Not considered, absent PAM |
| 3 | Intron 9 / *Usp22* | chr11:61048130-61048152 | AATAGCCAGGCGACCAAGCC | Yes (TGG) | Considered and assessed |
| 3 | Intergenic / 4933438B17Rik-4930572K03Rik | chr5:127207886-127207908 | AATAACCAGACCACCAATAT | Yes (GGG) | Not consider, intergenic |
| 3 | Intergenic / 1700083H02Rik-4930404H11Rik | chr12:71529591-71529613 | AAAAACCAGGCCACCAAGCT | Yes (GGG) | Not consider, intergenic |

| | | | | | |
|---|---|---|---|---|---|
| 3 | Intergenic / *Ambra1-Chrm4* | chr2:129054320-129054342 | AAGAACCAGGCGGCCAAGCT | Yes (TGG) | Not consider, intergenic |
| 3 | Intergenic / Slc20a1-A730036I17Rik | chr2:129054320-129054342 | AATACCCAGTCAACCAATCT | No (GGA) | Not considered, absent PAM |

** The nucleotides highlighted in red color represent the mismatches to the sgRNA sequence.*

*** The off-targets considered for further assessment are highlighted in grey color.*

**Supplementary Table S4.** Primers used for the assessment of the predicted off-targets.

| *Off-targets of sgRNA no.* | Location / Gene | *Off-target sequence* | *Primer sequences* |
|---|---|---|---|
| 1 | Intron 3 / Abcc8 | CCTAGCCTGGATTGGTCGCC | [Fw] CTTTCCTCCCCAGCATTCTCA<br>[Rv] GCCATCACCTCTGTGGTCTAC |
| 2 | Intron 9 / Usp22 | AATAGCCAGGCGACCAAGCC | [Fw] ATGAAGGGCGTCATGTCCAG<br>[Rv] GCCTGAGTAGGCTTGTGAGT |

**Supplementary Table S5.** Primers used for gene expression investigation of muscular maturation markers.

| *Gene symbol* | *Gene name* | *Ensembl Gene ID* | *Primer sequences* |
|---|---|---|---|
| Mef2c | Myocyte enhancer factor 2C | ENSMUSG00000005583 | [Fw] GCCGGACAAACTCAGACATT<br>[Rv] TGGGATGGTAACTGGCATCT |
| Dmd | Dystrophin | ENSMUSG00000045103 | [Fw] GTGGGAAGAAGTAGAGGACTGTT<br>[Rv] AGGTCTAGGAGGCGTTTTCC |
| Ap3d1 | Adaptor related protein complex 3 delta 1 subunit | ENSMUSG00000020198 | [Fw] TGAGAAGTTGGACTTCCGGC<br>[Rv] AAAGGCATCGCTGTAGCAGG |

**Supplementary Table S6.** List of MRM transitions and collision energies of nucleotide sugars and their isotopically labeled analogs. For all transitions reported in the table, we applied: polarity=negative; cell accelerator voltage=7; fragmentor=380.

| | Mass (amu) | Isomeric SMILES | Collision Energy | RT (min) |
|---|---|---|---|---|
| **UDP-Glucose** | | | | |
| **Precursor ion** | 565 | O=c1ccn([C@@H]2O[C@H](COP(=O)(O)OP(=O)(O)OC3O[C@H](CO)[C@@H](O)[C@H](O)[C@H]3O)[C@@H](O)[C@H]2O)c(=O)[nH]1 | | |
| **Product ion 1** | 403 | O=c1ccn([C@@H]2O[C@H](COP(=O)(O)OP(=O)([O-])O)[C@@H](O)[C@H]2O)c(=O)[nH]1 | 24 | 8.3 |
| **Product ion 2** | 385 | O=c1ccn([C@@H]2O[C@H](COP(=O)([O-])OP(=O)=O)[C@@H](O)[C@H]2O)c(=O)[nH]1 | 26 | 8.3 |
| **Product ion 3** | 323 | O=c1ccn([C@@H]2O[C@H](COP(=O)([O-])O)[C@@H](O)[C@H]2O)c(=O)[nH]1 | 22 | 8.3 |
| **Product ion 4** | 273 | O=P(=O)OP(=O)([O-])OC[C@H]1OC=C(O)[C@@H]1O | 36 | 8.3 |
| **Product ion 5** | 241 | O=P1([O-])OC2O[C@H](CO)[C@@H](O1)[C@H](O)[C@H]2O | 30.7 | 8.3 |
| **Product ion 6** | 158.9 | O=P(=O)OP(=O)([O-])O | 45.3 | 8.3 |
| **Product ion 7** | 97 | O=P([O-])(O)O | 51 | 8.3 |
| **Product ion 8** | 79 | O=P(=O)[O-] | 61.3 | 8.3 |
| **UDP-Galactose** | | | | |
| **Precursor ion** | 565 | O=c1ccn([C@@H]2O[C@H](COP(=O)(O)OP(=O)(O)OC3O[C@H](CO)[C@H](O)[C@H](O)[C@H]3O)[C@@H](O)[C@H]2O)c(=O)[nH]1 | | |
| **Product ion 1** | 403 | O=c1ccn([C@@H]2O[C@H](COP(=O)(O)OP(=O)([O-])O)[C@@H](O)[C@H]2O)c(=O)[nH]1 | 24 | 7.4 |
| **Product ion 2** | 385 | O=c1ccn([C@@H]2O[C@H](COP(=O)([O-])OP(=O)=O)[C@@H](O)[C@H]2O)c(=O)[nH]1 | 26 | 7.4 |

| | | | | |
|---|---|---|---|---|
| **Product ion 3** | 323 | O=c1ccn([C@@H]2O[C@H](COP(=O)([O-])O)[C@@H](O)[C@H]2O)c(=O)[nH]1 | 22 | 7.4 |
| **Product ion 4** | 273 | O=P(=O)OP(=O)([O-])OC[C@H]1OC=C(O)[C@@H]1O | 36 | 7.4 |
| **Product ion 5** | 241 | O=P1([O-])OC2O[C@H](CO)[C@H](O1)[C@H](O)[C@H]2O | 30.7 | 7.4 |
| **Product ion 6** | 158.9 | O=P(=O)OP(=O)([O-])O | 45.3 | 7.4 |
| **Product ion 7** | 97 | O=P([O-])(O)O | 51 | 7.4 |
| **Product ion 8** | 79 | O=P(=O)[O-] | 61.3 | 7.4 |
| **$^{13}C_5$-UDP-Glucose** | | | | |
| **Precursor ion** | 570.1 | O=c1ccn([13C@@H]2O[13C@H]([13CH2]OP(=O)(O)OP(=O)(O)OC3O[C@H](CO)[C@@H](O)[C@H](O)[C@H]3O)[13C@@H](O)[13C@H]2O)c(=O)[nH]1 | | |
| **Product ion 1** | 408 | O=c1ccn([13C@@H]2O[13C@H]([13CH2]OP(=O)(O)OP(=O)([O-])O)[13C@@H](O)[13C@H]2O)c(=O)[nH]1 | 24 | 8.3 |
| **Product ion 2** | 390 | O=c1ccn([13C@@H]2O[13C@H]([13CH2]OP(=O)([O-])OP(=O)=O)[13C@@H](O)[13C@H]2O)c(=O)[nH]1 | 26 | 8.3 |
| **Product ion 3** | 328 | O=c1ccn([13C@@H]2O[13C@H]([13CH2]OP(=O)([O-])O)[13C@@H](O)[13C@H]2O)c(=O)[nH]1 | 22 | 8.3 |
| **Product ion 4** | 278 | O=P(=O)OP(=O)([O-])O[13CH2][13C@H]1O[13CH]=[13C](O)[13C@@H]1O | 36 | 8.3 |
| **Product ion 5** | 241 | O=P1([O-])OC2O[C@H](CO)[C@@H](O1)[C@H](O)[C@H]2O | 30.7 | 8.3 |
| **Product ion 6** | 158.9 | O=P(=O)OP(=O)([O-])O | 45.3 | 8.3 |
| **Product ion 7** | 97 | O=P([O-])(O)O | 51 | 8.3 |
| **Product ion 8** | 79 | O=P(=O)[O-] | 61.3 | 8.3 |
| **$^{13}C_5$-UDP-Galactose** | | | | |
| **Precursor ion** | 570.1 | O=c1ccn([13C@@H]2O[13C@H]([13CH2]OP(=O)(O)OP(=O)(O)OC3O[C@H](CO)[C@H](O)[C@H](O)[C@H]3O)[13C@@H](O)[13C@H]2O)c(=O)[nH]1 | | |
| **Product ion 1** | 408 | O=c1ccn([13C@@H]2O[13C@H]([13CH2]OP(=O)(O)OP(=O)([O-])O)[13C@@H](O)[13C@H]2O)c(=O)[nH]1 | 24 | 7.4 |
| **Product ion 2** | 390 | O=c1ccn([13C@@H]2O[13C@H]([13CH2]OP(=O)([O-])OP(=O)=O)[13C@@H](O)[13C@H]2O)c(=O)[nH]1 | 26 | 7.4 |
| **Product ion 3** | 328 | O=c1ccn([13C@@H]2O[13C@H]([13CH2]OP(=O)([O-])O)[13C@@H](O)[13C@H]2O)c(=O)[nH]1 | 22 | 7.4 |
| **Product ion 4** | 278 | O=P(=O)OP(=O)([O-])O[13CH2][13C@H]1O[13CH]=[13C](O)[13C@@H]1O | 36 | 7.4 |
| **Product ion 5** | 241 | O=P1([O-])OC2O[C@H](CO)[C@H](O1)[C@H](O)[C@H]2O | 30.7 | 7.4 |
| **Product ion 6** | 158.9 | O=P(=O)OP(=O)([O-])O | 45.3 | 7.4 |
| **Product ion 7** | 97 | O=P([O-])(O)O | 51 | 7.4 |
| **Product ion 8** | 79 | O=P(=O)[O-] | 61.3 | 7.4 |
| **$^{13}C_6$-UDP-Glucose** | | | | |
| **Precursor ion** | 571.1 | O=c1ccn([C@@H]2O[C@H](COP(=O)(O)OP(=O)(O)O[13CH]3O[13C@H]([13CH2]O)[13C@@H](O)[13C@H](O)[13C@H]3O)[C@@H](O)[C@H]2O)c(=O)[nH]1 | | |
| **Product ion 1** | 403 | O=c1ccn([C@@H]2O[C@H](COP(=O)(O)OP(=O)([O-])O)[C@@H](O)[C@H]2O)c(=O)[nH]1 | 24 | 8.3 |
| **Product ion 2** | 385 | O=c1ccn([C@@H]2O[C@H](COP(=O)([O-])OP(=O)=O)[C@@H](O)[C@H]2O)c(=O)[nH]1 | 26 | 8.3 |
| **Product ion 3** | 273 | O=P(=O)OP(=O)([O-])OC[C@H]1OC=C(O)[C@@H]1O | 22 | 8.3 |
| **Product ion 4** | 323 | O=c1ccn([C@@H]2O[C@H](COP(=O)([O-])O)[C@@H](O)[C@H]2O)c(=O)[nH]1 | 36 | 8.3 |
| **Product ion 5** | 247 | O=P1([O-])O[13CH]2O[13C@H]([13CH2]O)[13C@@H](O1)[13C@H](O)[13C@H]2O | 30.7 | 8.3 |
| **Product ion 6** | 158.9 | O=P(=O)OP(=O)([O-])O | 45.3 | 8.3 |
| **Product ion 7** | 97 | O=P([O-])(O)O | 51 | 8.3 |
| **Product ion 8** | 79 | O=P(=O)[O-] | 61.3 | 8.3 |
| **$^{13}C_6$-UDP-Galactose** | | | | |
| **Precursor ion** | 571.1 | O=c1ccn([C@@H]2O[C@H](COP(=O)(O)OP(=O)(O)O[13CH]3O[13C@H]([13CH2]O)[13C@H](O)[13C@H](O)[13C@H]3O)[C@@H](O)[C@H]2O)c(=O)[nH]1 | | |
| **Product ion 1** | 403 | O=c1ccn([C@@H]2O[C@H](COP(=O)(O)OP(=O)([O-])O)[C@@H](O)[C@H]2O)c(=O)[nH]1 | 24 | 7.4 |
| **Product ion 2** | 385 | O=c1ccn([C@@H]2O[C@H](COP(=O)([O-])OP(=O)=O)[C@@H](O)[C@H]2O)c(=O)[nH]1 | 26 | 7.4 |
| **Product ion 3** | 323 | O=c1ccn([C@@H]2O[C@H](COP(=O)([O-])O)[C@@H](O)[C@H]2O)c(=O)[nH]1 | 22 | 7.4 |

| | | | | |
|---|---|---|---|---|
| **Product ion 4** | 273 | O=P(=O)OP(=O)([O-])OC[C@H]1OC=C(O)[C@@H]1O | 36 | 7.4 |
| **Product ion 5** | 247 | O=P1([O-])O[13CH]2O[13C@H]([13CH2]O)[13C@H](O1)[13C@H](O)[13C@H]2O | 30.7 | 7.4 |
| **Product ion 6** | 158.9 | O=P(=O)OP(=O)([O-])O | 45.3 | 7.4 |
| **Product ion 7** | 97 | O=P([O-])(O)O | 51 | 7.4 |
| **Product ion 8** | 79 | O=P(=O)[O-] | 61.3 | 7.4 |
| **$^{13}C_{11}$-UDP-Glucose** | | | | |
| **Precursor ion** | 576.1 | O=c1ccn([13C@@H]2O[13C@H]([13CH2]OP(=O)(O)OP(=O)(O)O[13CH]3O[13C@H]([13CH2]O)[13C@@H](O)[13C@H](O)[13C@H]3O)[13C@@H](O)[13C@H]2O)c(=O)[nH]1 | | |
| **Product ion 1** | 408 | O=c1ccn([13C@@H]2O[13C@H]([13CH2]OP(=O)(O)OP(=O)([O-)O)[13C@@H](O)[13C@H]2O)c(=O)[nH]1 | 24 | 8.3 |
| **Product ion 2** | 390 | O=c1ccn([13C@@H]2O[13C@H]([13CH2]OP(=O)([O-])OP(=O)=O)[13C@@H](O)[13C@H]2O)c(=O)[nH]1 | 26 | 8.3 |
| **Product ion 3** | 328 | O=c1ccn([13C@@H]2O[13C@H]([13CH2]OP(=O)([O-])O)[13C@@H](O)[13C@H]2O)c(=O)[nH]1 | 22 | 8.3 |
| **Product ion 4** | 278 | O=P(=O)OP(=O)([O-])O[13CH2][13C@H]1O[13CH]=[13C](O)[13C@@H]1O | 36 | 8.3 |
| **Product ion 5** | 247 | O=P1([O-])O[13CH]2O[13C@H]([13CH2]O)[13C@@H](O1)[13C@H](O)[13C@H]2O | 30.7 | 8.3 |
| **Product ion 6** | 158.9 | O=P(=O)OP(=O)([O-])O | 45.3 | 8.3 |
| **Product ion 7** | 97 | O=P([O-])(O)O | 51 | 8.3 |
| **Product ion 8** | 79 | O=P(=O)[O-] | 61.3 | 8.3 |
| **$^{13}C_{11}$-UDP-Galactose** | | | | |
| **Precursor ion** | 576.1 | O=c1ccn([13C@@H]2O[13C@H]([13CH2]OP(=O)(O)OP(=O)(O)O[13CH]3O[13C@H]([13CH2]O)[13C@H](O)[13C@H](O)[13C@H]3O)[13C@@H](O)[13C@H]2O)c(=O)[nH]1 | | |
| **Product ion 1** | 408 | O=c1ccn([13C@@H]2O[13C@H]([13CH2]OP(=O)(O)OP(=O)([O-])O)[13C@@H](O)[13C@H]2O)c(=O)[nH]1 | 24 | 7.4 |
| **Product ion 2** | 390 | O=c1ccn([13C@@H]2O[13C@H]([13CH2]OP(=O)([O-])OP(=O)=O)[13C@@H](O)[13C@H]2O)c(=O)[nH]1 | 26 | 7.4 |
| **Product ion 3** | 328 | O=c1ccn([13C@@H]2O[13C@H]([13CH2]OP(=O)([O-])O)[13C@@H](O)[13C@H]2O)c(=O)[nH]1 | 22 | 7.4 |
| **Product ion 4** | 278 | O=P(=O)OP(=O)([O-])O[13CH2][13C@H]1O[13CH]=[13C](O)[13C@@H]1O | 36 | 7.4 |
| **Product ion 5** | 247 | O=P1([O-])O[13CH]2O[13C@H]([13CH2]O)[13C@H](O1)[13C@H](O)[13C@H]2O | 30.7 | 7.4 |
| **Product ion 6** | 158.9 | O=P(=O)OP(=O)([O-])O | 45.3 | 7.4 |
| **Product ion 7** | 97 | O=P([O-])(O)O | 51 | 7.4 |
| **Product ion 8** | 79 | O=P(=O)[O-] | 61.3 | 7.4 |

**Supplementary Table S7.** List of MRM transitions and collision energies of hexose phosphates and their isotopically labeled analogs. For all transitions reported in the table, we applied: polarity=negative; cell accelerator voltage=7; fragmentor=380.

| | Mass (amu) | Isomeric SMILES | Collision Energy | RT (min) |
|---|---|---|---|---|
| **Glucose 1-phosphate** | | | | |
| **Precursor ion** | 259 | O=P(O)(O)O[C@@H]1O[C@H](CO)[C@@H](O)[C@H](O)[C@H]1O | | |
| **Product ion 1** | 241 | O=P1([O-])O[C@@H]2O[C@H](CO)[C@@H](O)[C@H](O)[C@H]2O1 | 9 | 10.5 |
| **Product ion 2** | 139 | O=CCOP(=O)([O-])O | 10.83 | 10.5 |
| **Product ion 3** | 97 | O=P([O-])(O)O | 13.25 | 10.5 |
| **Product ion 4** | 79 | O=P(=O)[O-] | 50.5 | 10.5 |
| **Glucose 6-phosphate** | | | | |
| **Precursor ion** | 259 | O=C[C@H](O)[C@@H](O)[C@H](O)[C@H](O)COP(=O)(O)O | | |
| **Product ion 1** | 199 | O=C[C@H](O)[C@H](O)COP(=O)([O-])O | 7 | 8.5 |
| **Product ion 2** | 169 | O=C[C@H](O)COP(=O)([O-])O | 5.8 | 8.5 |

| | | | | |
|---|---|---|---|---|
| **Product ion 3** | 139 | O=CCOP(=O)([O-])O | 10.83 | 8.5 |
| **Product ion 4** | 97 | O=P([O-])(O)O | 13.25 | 8.5 |
| **Product ion 5** | 79 | O=P(=O)[O-] | 50.5 | 8.5 |
| **Galactose 1-phosphate** | | | | |
| **Precursor ion** | 259 | O=P(O)(O)O[C@H]1O[C@H](CO)[C@H](O)[C@H](O)[C@H]1O | | |
| **Product ion 1** | 241 | O=P1([O-])O[C@H]2O[C@H](CO)[C@H](O1)[C@H](O)[C@H]2O | 9 | 9.5 |
| **Product ion 2** | 139 | O=CCOP(=O)([O-])O | 10.83 | 9.5 |
| **Product ion 3** | 97 | O=P([O-])(O)O | 13.25 | 9.5 |
| **Product ion 4** | 79 | O=P(=O)[O-] | 50.5 | 9.5 |
| **$^{13}C_5$-Glucose 1-phosphatealactose** | | | | |
| **Precursor ion** | 265 | O=P(O)(O)O[13C@@H]1O[13C@H]([13CH2]O)[13C@@H](O)[13C@H](O)[13C@H]1O | | |
| **Product ion 1** | 247 | O=P1([O-])O[13C@@H]2O[13C@H]([13CH2]O)[13C@@H](O)[13C@H](O)[13C@H]2O1 | 9 | 10.5 |
| **Product ion 2** | 141 | O=[13CH][13CH2]OP(=O)([O-])O | 10.83 | 10.5 |
| **Product ion 3** | 97 | O=P([O-])(O)O | 13.25 | 10.5 |
| **Product ion 4** | 79 | O=P(=O)[O-] | 50.5 | 10.5 |
| **$^{13}C_6$-Glucose 6-phosphate** | | | | |
| **Precursor ion** | 265 | O=[13CH][13C@H](O)[13C@@H](O)[13C@H](O)[13C@H](O)[13CH2]OP(=O)(O)O | | |
| **Product ion 1** | 203 | O=[13CH][13C@H](O)[13C@H](O)[13CH2]OP(=O)([O-])O | 7 | 8.5 |
| **Product ion 2** | 172 | O=[13CH][13CH2]OP(=O)([O-])O | 5.8 | 8.5 |
| **Product ion 3** | 141 | O=[13CH][13CH2]OP(=O)([O-])O | 10.83 | 8.5 |
| **Product ion 4** | 97 | O=P([O-])(O)O | 13.25 | 8.5 |
| **Product ion 5** | 79 | O=P(=O)[O-] | 50.5 | 8.5 |
| **$^{13}C_6$-Galactose 1-phosphate** | | | | |
| **Precursor ion** | 265 | O=P(O)(O)O[13C@H]1O[13C@H]([13CH2]O)[13C@H](O)[13C@H](O)[13C@H]1O | | |
| **Product ion 1** | 247 | O=P1([O-])O[13CH2][13C@H]2O[13C@H](O1)[13C@H](O)[13C@@H](O)[13C@H]2O | 9 | 9.5 |
| **Product ion 2** | 141 | O=[13CH][13CH2]OP(=O)([O-])O | 10.83 | 9.5 |
| **Product ion 3** | 97 | O=P([O-])(O)O | 13.25 | 9.5 |
| **Product ion 4** | 79 | O=P(=O)[O-] | 50.5 | 9.5 |

**Supplementary Table S8.** Titration of optimized FCCP concentrations used for the mitochondrial stress test (MST).

| | | *Wild-type line* | *Pgm1-KO clone 1* |
|---|---|---|---|
| *Myoblasts* | **Treatment** | **Optimal [FCCP]** | **Optimal [FCCP]** |
| | 5.5 mM Glc | 0.5 µM | 1 µM |
| | 5.5 mM Gal | 2 µM | 2 µM |
| | 5 mM Glc + 0.5 mM Gal | 2 µM | 3 µM |
| | 5 mM Glc + 5 mM Gal | 2 µM | 2 µM |
| *Myotubes* | **Treatment** | **Optimal [FCCP]** | **Optimal [FCCP]** |
| | 5.5 mM Glc | 2 µM | 2 µM |
| | 5.5 mM Gal | 1 µM | 1 µM |
| | 5 mM Glc + 0.5 mM Gal | 2 µM | 2 µM |
| | 5 mM Glc + 5 mM Gal | 2 µM | 1 µM |

**Supplementary Figure S1. Sequencing results of the CRISPR/Cas9 target sequence on *Pgm1* gene.**

[**a**] Target sequence in *Pgm1* wild-type gene via Sanger sequencing. [**b**] Target sequence of *Pgm1* gene in *Pgm1*-KO clone 1. pGEM sequencing revealed that the clone is a compound heterozygous, with a 1bp insertion causing frameshift on Allele 1, and a 15bp deletion on Allele 2 causing an in-frame 5aa deletion. [**c**] Target sequence of *Pgm1* gene in *Pgm1*-KO clone 2. The sequencing revealed that the clone present a homozygous 1bp insertion. The sequence variants [**b,c**] are reported as genomic, coding DNA and protein sequences, according to ACMG nomenclature guidelines.

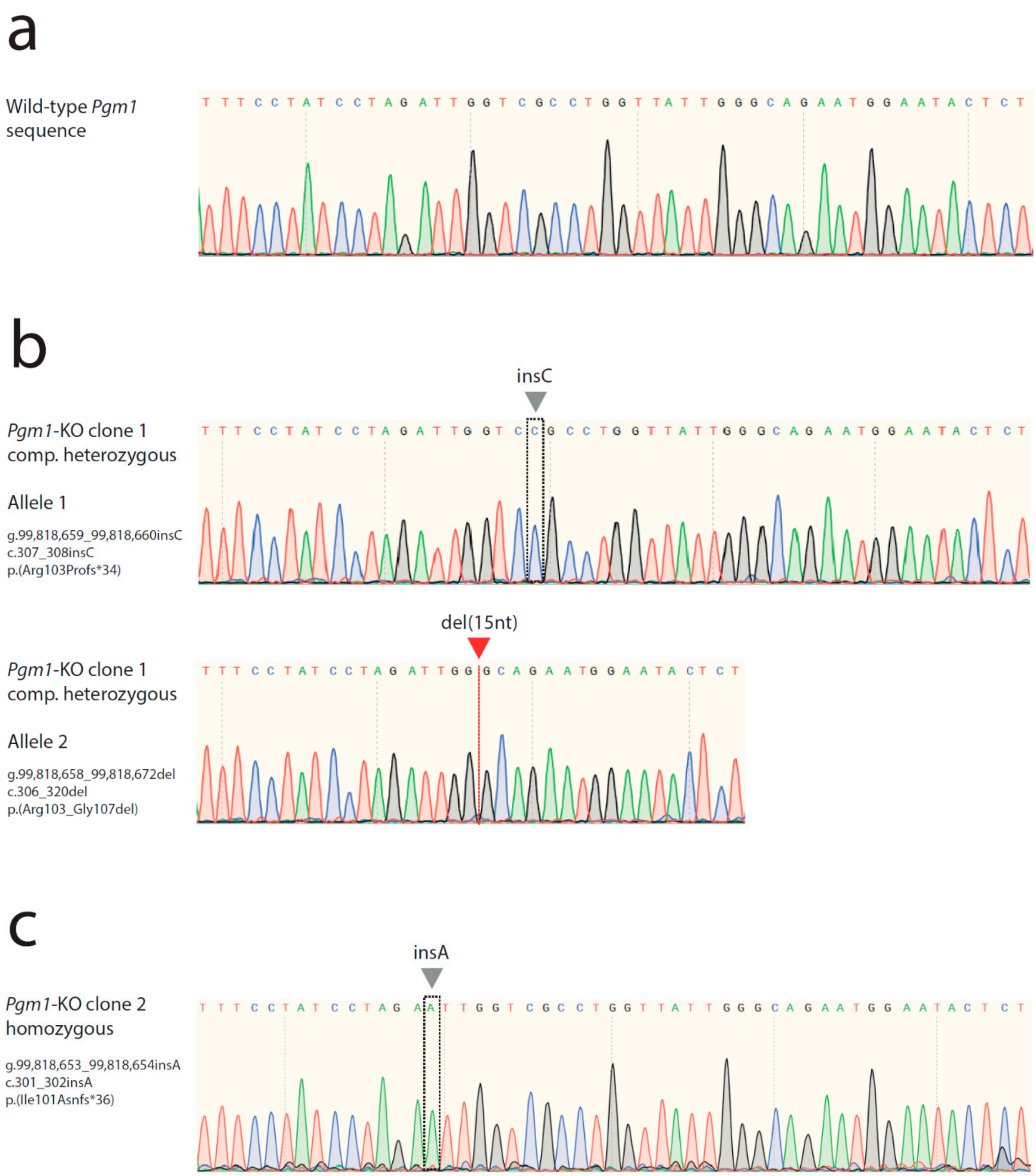

**Supplementary Figure S2. Results of the off-target sequencing.**

Results from Sanger sequencing of the predicted off-targets for sgRNA 1 (**a**) and sgRNA 2 (**b**). Asterisks (*) indicate the position of the mismatches. Sanger sequencing did not reveal any off-target effect in these clones for the regions assessed.

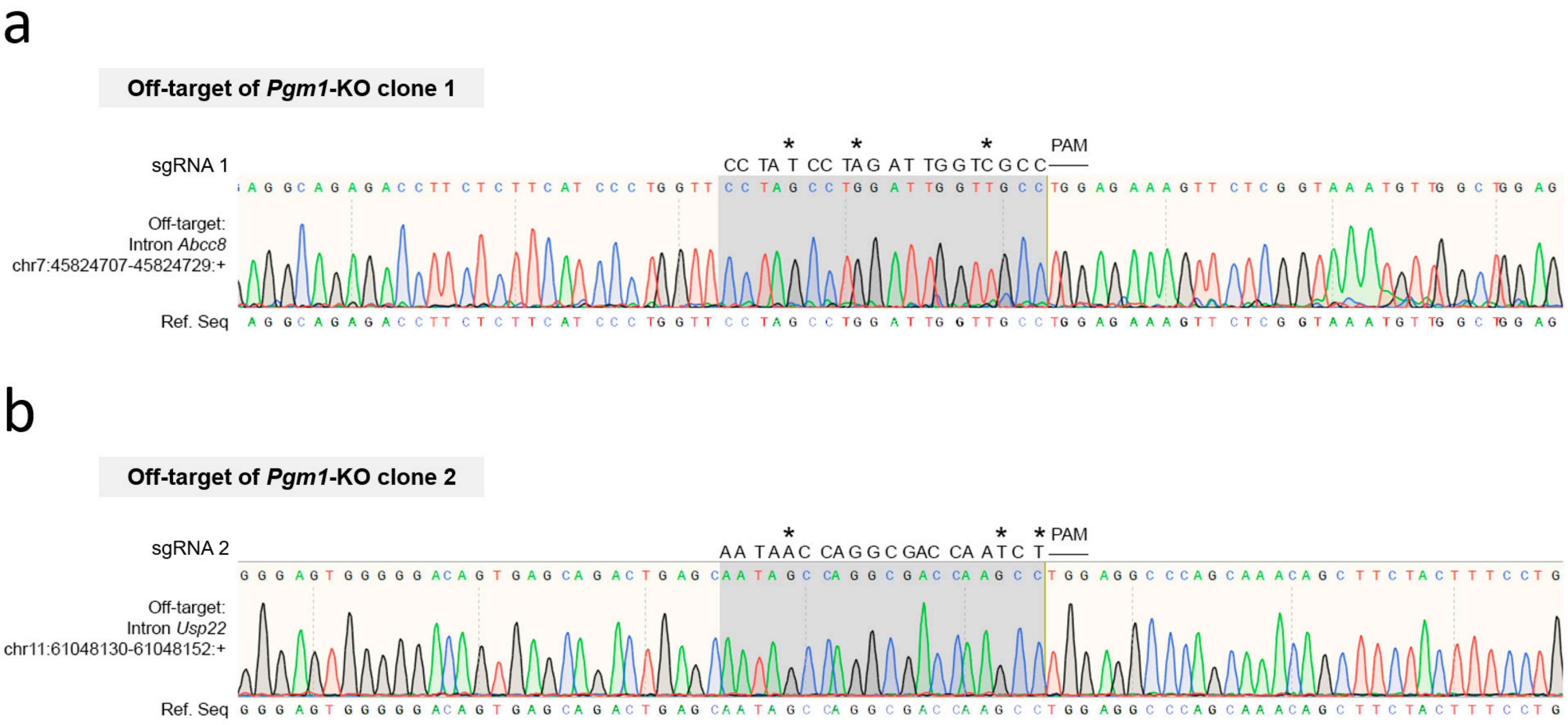

**Supplementary Figure S3. Wild-type and KO lines during differentiation (supplementary pictures).**
Brightfield pictures of wild-type (WT, panel **a**) and KO clones 1 (KO-1, panel **b**) and 2 (KO-2, panel **c**) at day -1 (D-1, corresponding to myoblasts 24 hours before starting differentiation), day 3 (D3, intermediate state) and day 7 (D7, myotube state). These cells have been differentiated in commercial differentiation medium containing 25 mM glucose (see **Table 2**).

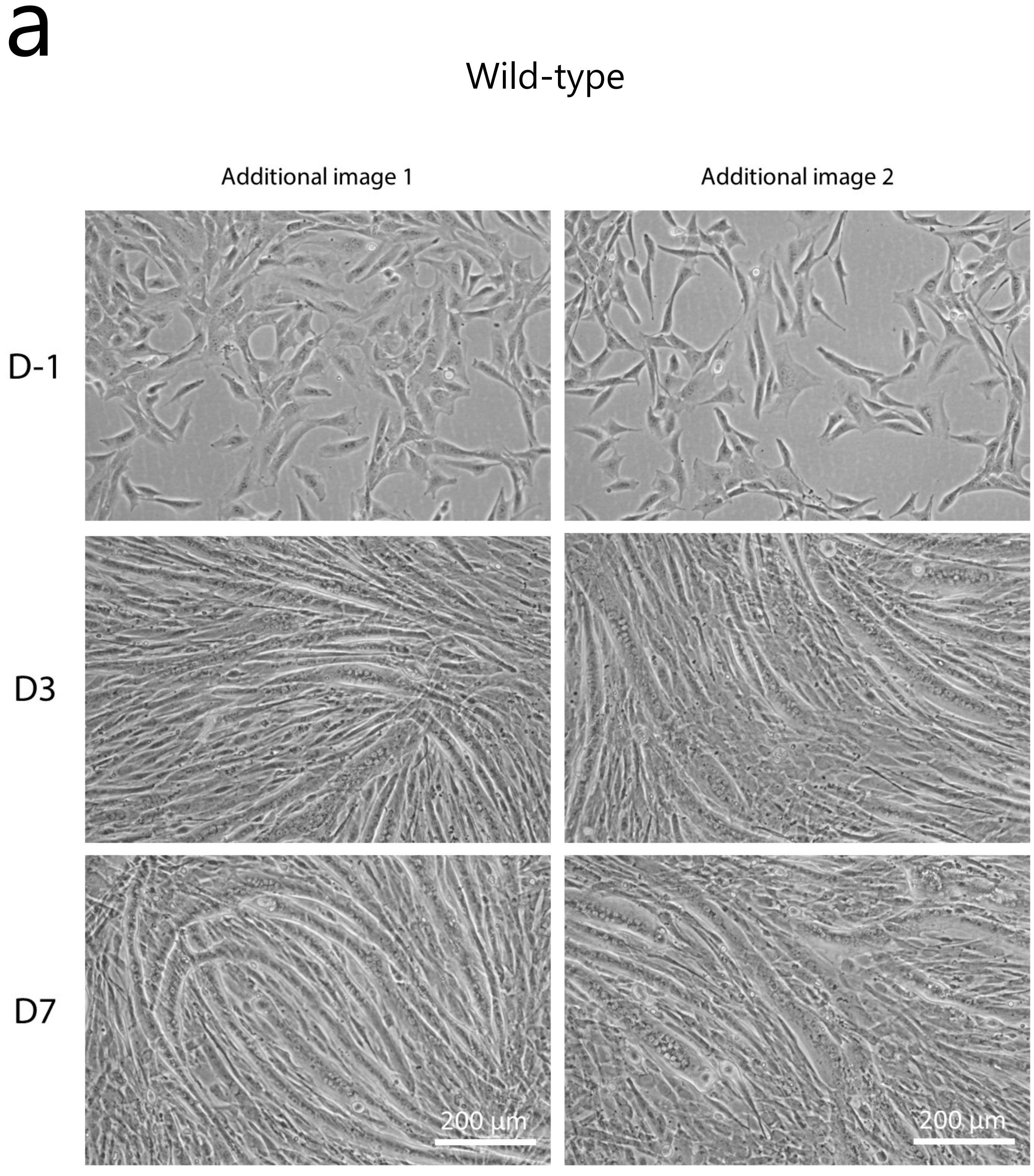

b

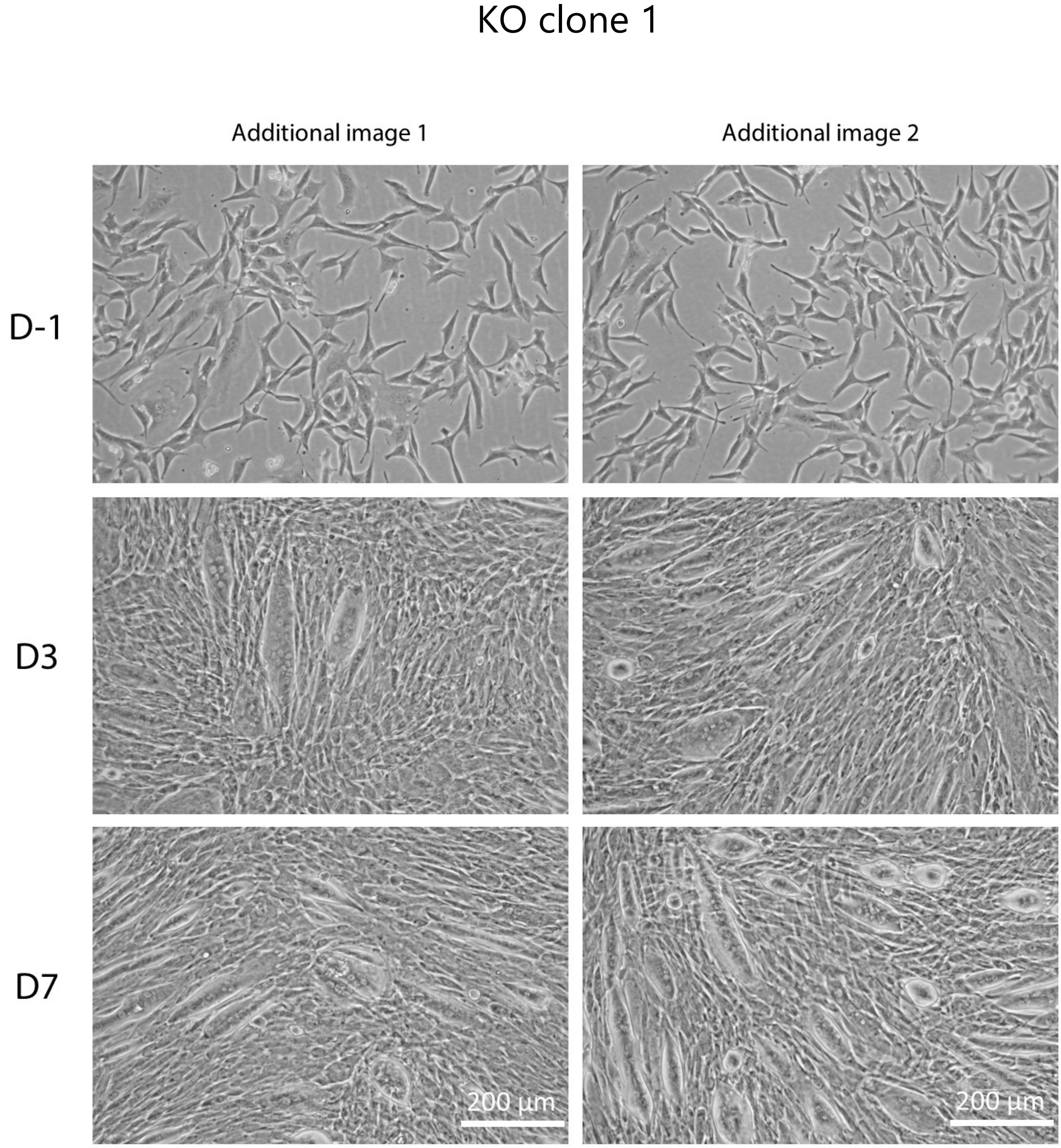
KO clone 1
Additional image 1
Additional image 2
D-1
D3
D7
200 µm
200 µm

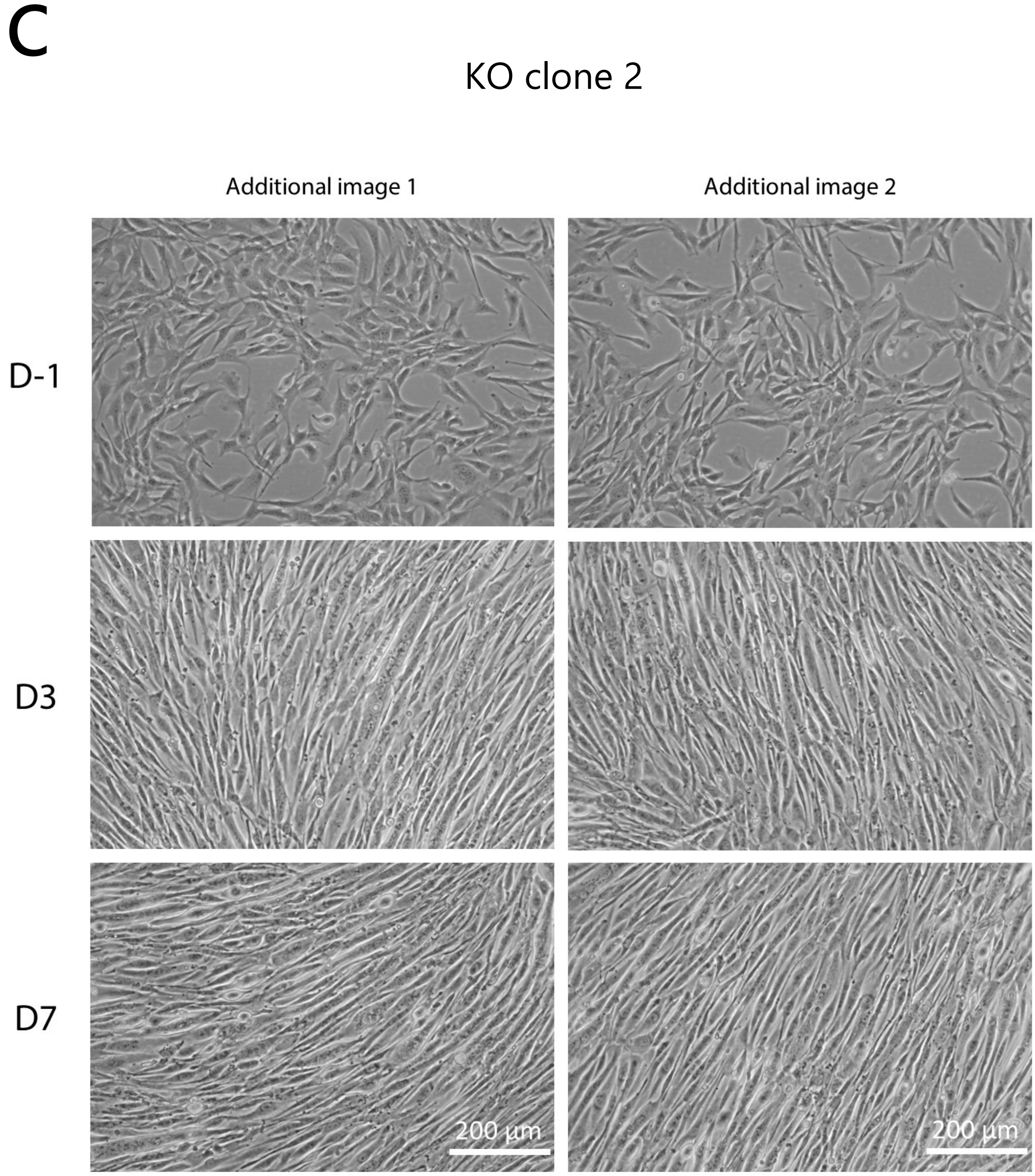
C
KO clone 2
Additional image 1
Additional image 2
D-1
D3
D7
200 µm
200 µm

**Supplementary Figure S4. Wild-type and KO myotubes differentiated in in presence of galactose.**

Brightfield pictures of wild-type (WT) and KO clones (KO-1, KO-2) differentiated in four different differentiation media, each with a different sugar content (see also **Table 2**): (1) medium supplemented with 25 mM glucose (control); (2) medium supplemented with 25 mM galactose; (3) medium supplemented with 20 mM glucose and 2 mM galactose; (4) medium supplemented with 12.5 mM glucose and 12.5 mM galactose.

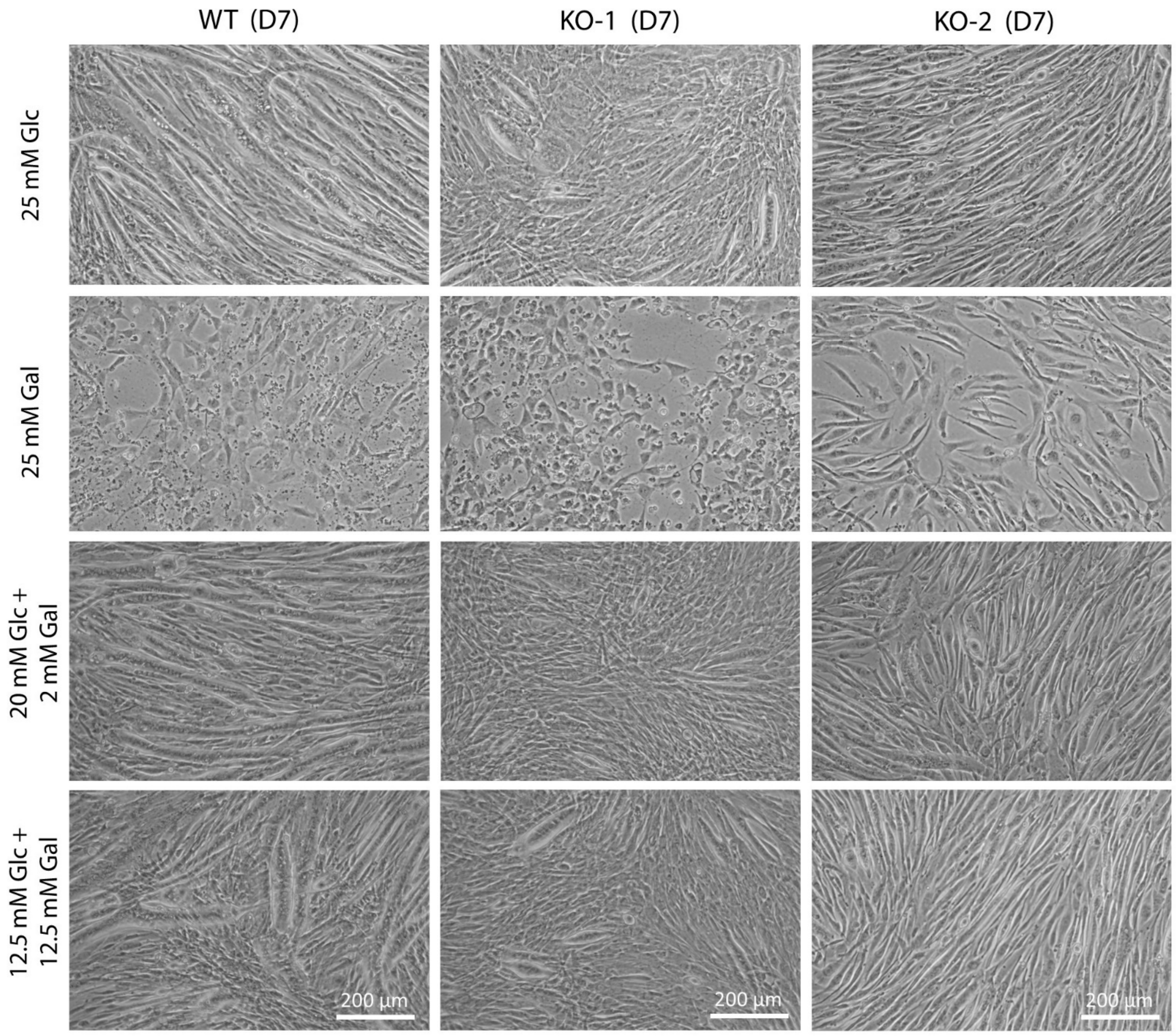

**Supplementary Figure S5. Gene expression of *Mef2c* and *Dmd* in myotubes cultured in presence of galactose.** Bar charts of the relative expression of myogenic maturation markers *Mef2c* and *Dmd* during differentiation (day 3, D3) and at myotube state (day 7, D7) in the presence of glucose and/or galactose in the differentiation medium (combinations reported in figure and in **Table 2**). The gene expression (expressed as fold change) is normalized on the expression of the house-keeping gene *Ap3d1* at timepoint 0. Error bars represent the standard deviation of $2^{-\Delta\Delta Cq}$ (n=3). Statistical significant was tested with one-way ANOVA with Bonferroni post hod correction for multiple comparrisons (** p < 0.01, ***p < 0.001, ****p< 0.0001).

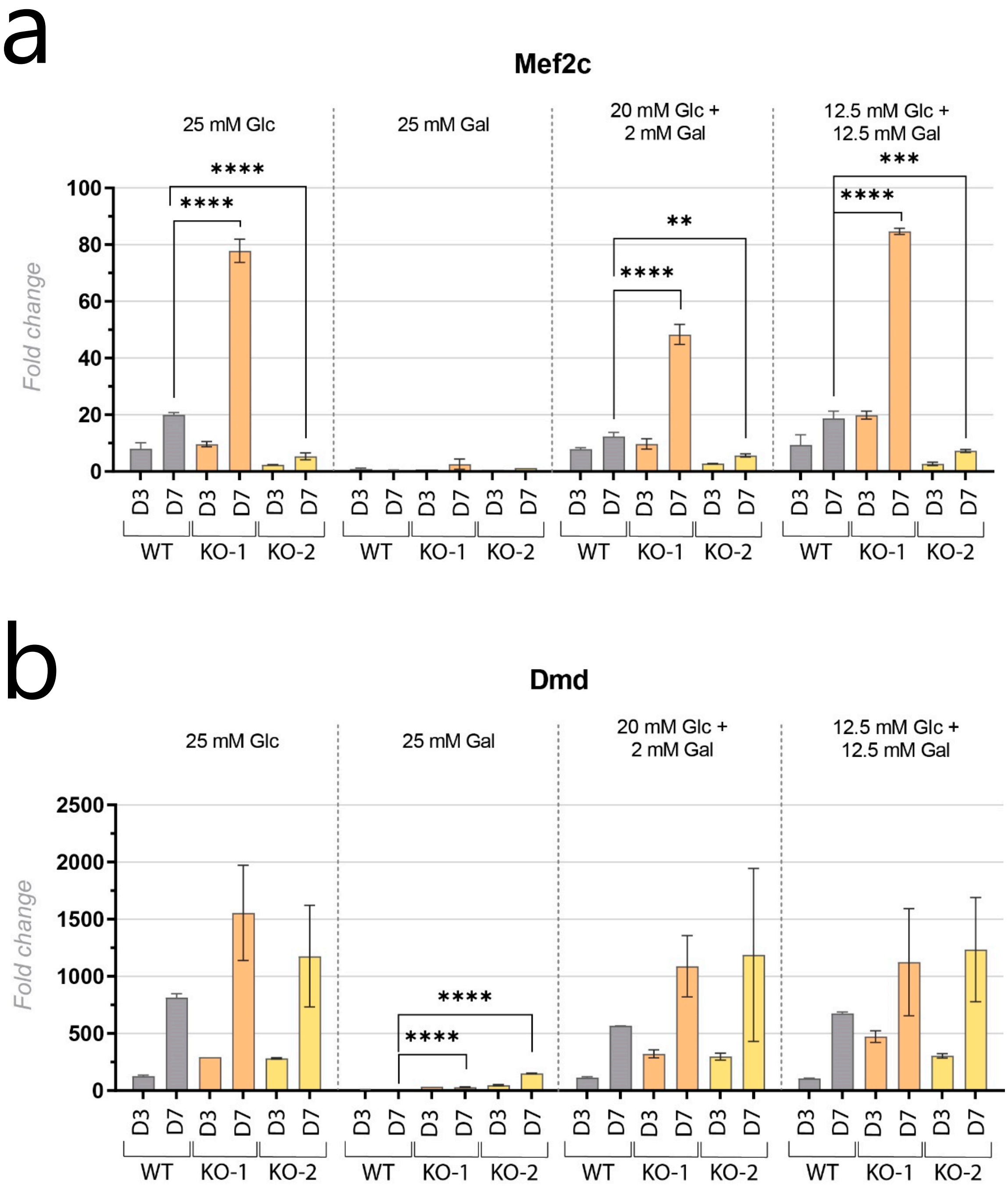

**Supplementary Figure S6. Non-mitochondrial respiration, proton leak and maximal respiratory capacity in wild-type and *Pgm1*-KO myoblasts and myotubes under galactose treatment.**

Proton leak indicates the remaining basal respiration not coupled to ATP production. Proton leak can be a sign of mitochondrial damage or can be used as a mechanism to regulate the mitochondrial ATP production. In (**a**) the proton leak is significantly lower in *Pgm1*-KO clone 1 myoblasts in all conditions except the condition without galactose (5.5. mM Glc). In (**b**), the proton leas is significantly lower in *Pgm1*-KO myotubes compared to wild-type myotubes in all conditions, except in the condition based on only galactose (5.5 mM Gal). Maximal respiration indicates the maximal OCR attained by adding the uncoupler FCCP, which pushes the respiratory chain to operate at maximum capacity and thus induces the maximum level of oxygen consumption achievable by the cell. The meet the metabolic challenge imposed by FCCP action, the cell is pushed towards rapid oxidation of substrates (sugars, fats, and amino acids). In (**a**), *Pgm1*-KO myoblasts display a significantly lower maximal respiratory capacity than wild-type myoblasts in all conditions. In (**b**), the only significant difference between the maximal respiratory capacity in wild-type myotubes and *Pgm1*-KO myotubes is observed in the condition based on only galactose feeding (5.5 mM Gal). Non-mitochondrial respiration indicates the oxygen consumption that persists due to a subset of cellular enzymes that continue to consume oxygen after the addition of rotenone and antimycin A. In (**a**), the non-mitochondrial respiration is mostly significantly lower in *Pgm1*-KO myoblasts when compared to wild-type myoblasts, with the only exception of the condition based on only galactose feeding (5.5 mM Gal). On the contrary, in (**b**) the only significant difference between the two cell lines is seen in the condition based on only galactose feeding. Data are expressed on OCR normalized on citrate synthase activity (CS). Statistical significance was calculated via two-way ANOVA with Bonferroni correction (*$p < 0.05$, ** $p < 0.01$, ***$p < 0.001$, ****$p < 0.0001$). The widest line in each point cloud represents the mean, while the vertical bar represents the standard deviation of the mean.

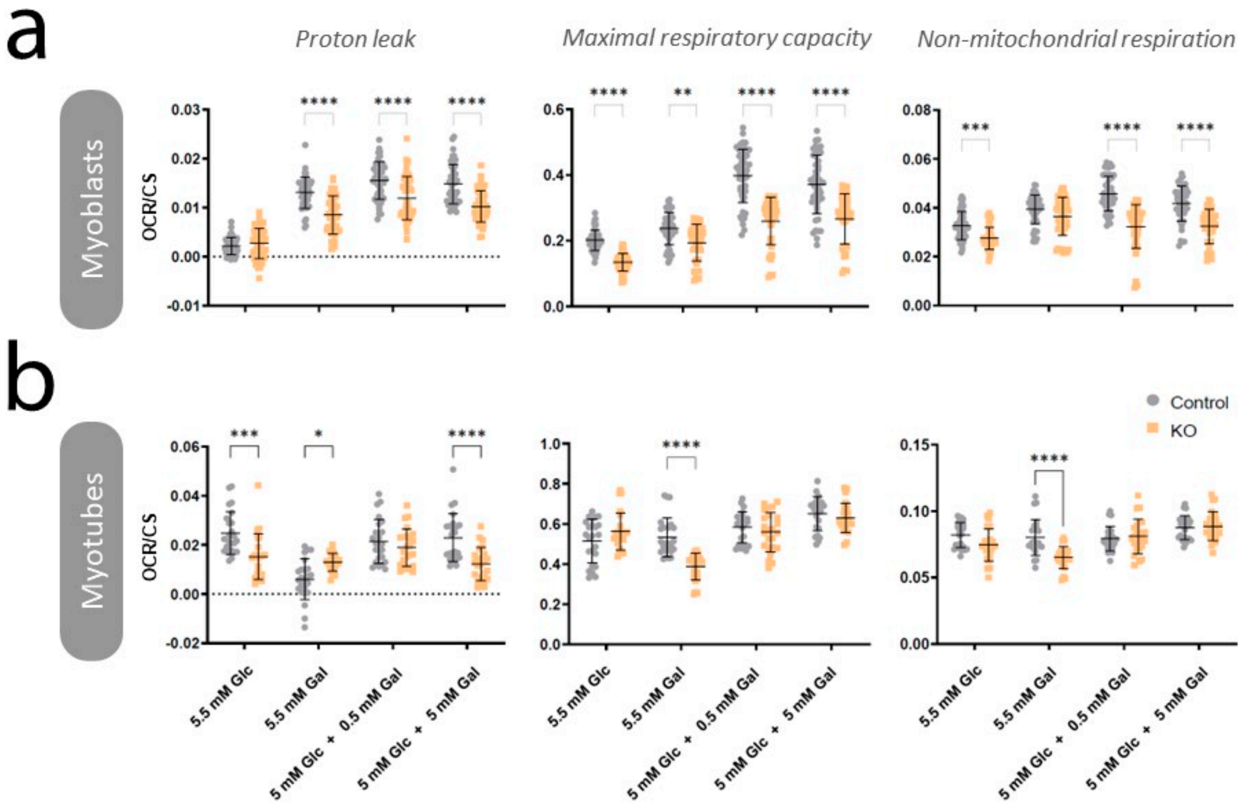

Supplement: Supplementary file 1 [file ijms-24-08247-s001.zip › Supplementary Figures & Tables.pdf]
